# Surveillance of potential hosts and vectors of scrub typhus in Taiwan

**DOI:** 10.1186/s13071-015-1221-7

**Published:** 2015-12-01

**Authors:** Chi-Chien Kuo, Pei-Lung Lee, Chun-Hsung Chen, Hsi-Chieh Wang

**Affiliations:** Department of Life Science, National Taiwan Normal University, Taipei, Taiwan; Center for Research, Diagnostics and Vaccine Development, Centers for Disease Control, Ministry of Health and Welfare, Taipei, No. 161, Kun-Yang Street, Taipei, 11561 Taiwan

**Keywords:** Hosts, *Leptotrombidium*, *Orientia tsutsugamushi*, *Rattus*, Scrub typhus, Taiwan, Vectors

## Abstract

**Background:**

Scrub typhus is a lethal infectious disease vectored by larval trombiculid mites (i.e. chiggers) infected with *Orientia tsutsugamushi* (OT) and recent decades have witnessed an emergence of scrub typhus in several countries. Identification of chigger species and their vertebrate hosts is fundamental for the assessment of human risks to scrub typhus under environmental changes, but intensive and extensive survey of chiggers and their hosts is still lacking in Taiwan.

**Methods:**

Chiggers were collected from shrews and rodents in nine counties of Taiwan and were assayed for OT infections with nested polymerase chain reaction (PCR). PCR products were further sequenced to reveal probable OT strains. Rodents were assessed for OT exposure by immunofluorescent antibody assay. Lastly, incidence rate of scrub typhus in each county was associated with loads and prevalence of chigger infestations, seropositivity rate in rodents, and OT positivity rate in chiggers.

**Results:**

*Rattus losea* was the most abundant (48.7 % of 1,285 individuals) and widespread (occurred in nine counties) small mammal species and hosted the majority of chiggers (76.4 % of 128,520 chiggers). *Leptotrombidium deliense* was the most common (64.9 % of all identified chiggers) and widespread (occurred in seven counties) chigger species but was replaced by *Leptotrombidium pallidum* or *Leptotrombidium scutellare* during the cold seasons in two counties (Matsu and Kinmen) where winter temperatures were lower than other study sites. Seropositivity rate for OT exposure in 876 assayed rodents was 43.0 % and OT positivity rate in 347 pools of chiggers was 55.9 %, with 15 OT strains identified in the 107 successfully sequenced samples. Incidence rate of scrub typhus was positively correlated with chigger loads, prevalence of chigger infestations, seropositivity rate but not OT positivity rate in chiggers.

**Conclusions:**

Our study reveals *R. losea* as the primary host for chiggers and there exists a geographical and seasonal variation in chigger species in Taiwan. It also emphasizes the importance of recognition of chigger vectors and their vertebrate hosts for a better prediction of human risks to scrub typhus under rapid environmental changes.

## Background

Scrub typhus is a potentially severe febrile disease transmitted by trombiculid mites infected with rickettsia *Orientia tsutsugamushi* (OT). Prevalent mainly along the western Pacific, about one million human cases occur each year and an estimated one billion people are at risk of this disease [1–3]. Except for parts of Asia, Australia, and Oceania where scrub typhus is traditionally endemic, suspected human cases have also been reported in Africa (Cameroon) and South America (Chile) [4, 5]. In addition, *Orientia*-like bacteria have been detected in rodents in France and Senegal [6]. In recent decades, several countries have experienced a great increase in the incidence of scrub typhus, including South Korea and China [7, 8]. In fact, scrub typhus is one of the most severely neglected diseases around the world [9, 10].

Among the different life stages of trombiculid mites, only larvae are parasitic, with rodents as the primary hosts and humans as the accidental hosts; nymphs and adults are free living in the soil, feeding mainly on arthropods [11]. Larval trombiculid mites (so called chiggers) are thus of significant medical importance because of their potential for transmitting scrub typhus to humans. Identification of chigger species is particularly fundamental for the evaluation of human risks because only a subset of chigger species, (mostly the genus *Leptotrombidium* [12]) are responsible for transmitting scrub typhus. Furthermore, even among the genus *Leptotrombidium*, chigger species vary in the seasonal occurrence and likely also virulence in the OT strains they carry. For instance, in Japan, *Leptotrombidium akamushi* occurs mainly in summer while *Leptotrombidium pallidum* and *Leptotrombidium scutellare* appear primarily from fall to early spring [11]. Besides, OT strains transmitted by *L. akamushi* and *L. pallidum* could be severe; in comparison, *L. scutellare*-borne OT might be relatively mild, although chigger-OT strain relationship is not fully understood [13, 14]. Recognition of chigger species is also essential for the elucidation of mechanisms underlying a potential surge in human incidence after global warming: such heighted risks could be due to an increase in chigger abundance or alternatively, the consequence of a change in chigger species. Similarly, when investigating environmental associations with spatial variation in incidence of scrub typhus (e.g. [15, 16]), what is actually modeled is how environments influence abundance of chiggers, which in turn determine human risks to scrub typhus. An implicit assumption for such investigation is that chiggers at different locations respond to environments identically, which might not be true when the primary vector species vary geographically. This can be exemplified, as mentioned above, by the difference in seasonal occurrence of *L. akamushi* versus *L. pallidum* or *L. scutellare*, which is more abundant at higher and lower temperatures, respectively [11].

Rodents are the main hosts of chiggers (as food resources) although their role in sustaining OT transmission (i.e. as a reservoir) requires further clarification [9, 11]. Rodents are thus indispensable for the survival of chiggers and play a key role in the transmission of scrub typhus. Because intensity of chigger infestations, even feeding success of chiggers, could vary considerably with rodent species [17, 18], it is imperative to recognize the primary host species. This will help predict whether a change in land use might enhance the risks to scrub typhus by favouring the survival of main rodent host species. For example, a transition to agricultural cultivation might increase the number of chiggers if their principal rodent hosts also flourish in agricultural fields. A good knowledge on the relative importance of rodent species might also assist the forecast of human health outcomes after an invasion of exotic rodent species [19].

In Taiwan, studies on vectors of scrub typhus are limited to a few places and no systematic survey has ever been implemented. *Leptotrombidium deliense* was found to be the primary chigger species in Penghu (Pescadores Islands, [20]) and *Leptotrombidium imphalum* was the most common species in Hualien [17]. In Kinmen Islands, occurrence of chigger species varies with seasons, with *L. deliense* as the main species in summer and *L. scutellare* the principal species in winter [21]. These sporadic studies suggest a geographical variation in chigger species and call for a more extensive survey of chiggers in Taiwan. Similarly, primary rodent hosts of chiggers have only been reported in a few localities in Taiwan, including Penghu, Kinmen, and Hualien [17, 21, 22]. Hosts of chiggers were surveyed in 15 counties in Taiwan but only an overall status was briefly reported; significance of local host species were only documented in one county [23]. Recently, hosts of chiggers were studied in six counties in Taiwan but only prevalence of chigger infestation (i.e. whether or not a host was infested with chiggers) was reported [24]; knowledge on the intensity of chigger infestation (i.e. the number of chiggers infested in each host) was still lacked for most regions of Taiwan.

In this study, chiggers and their associated hosts were systematically surveyed in different parts of Taiwan. In addition, we reported variation in seropositivity rate of OT infections among host species and geographical regions. Positivity rate of OT infections in chiggers and the probable OT strains were also studied. Lastly, we investigated whether geographic variation in human incidence of scrub typhus was correlated with prevalence and intensity of chigger infestations, OT seropositivity rate in rodent hosts, and OT positivity rate in chiggers. To our knowledge, this is the most extensive and comprehensive study on hosts and vectors of scrub typhus in Taiwan.

## Methods

### Study sites and small mammal trapping

**Fig 1:**
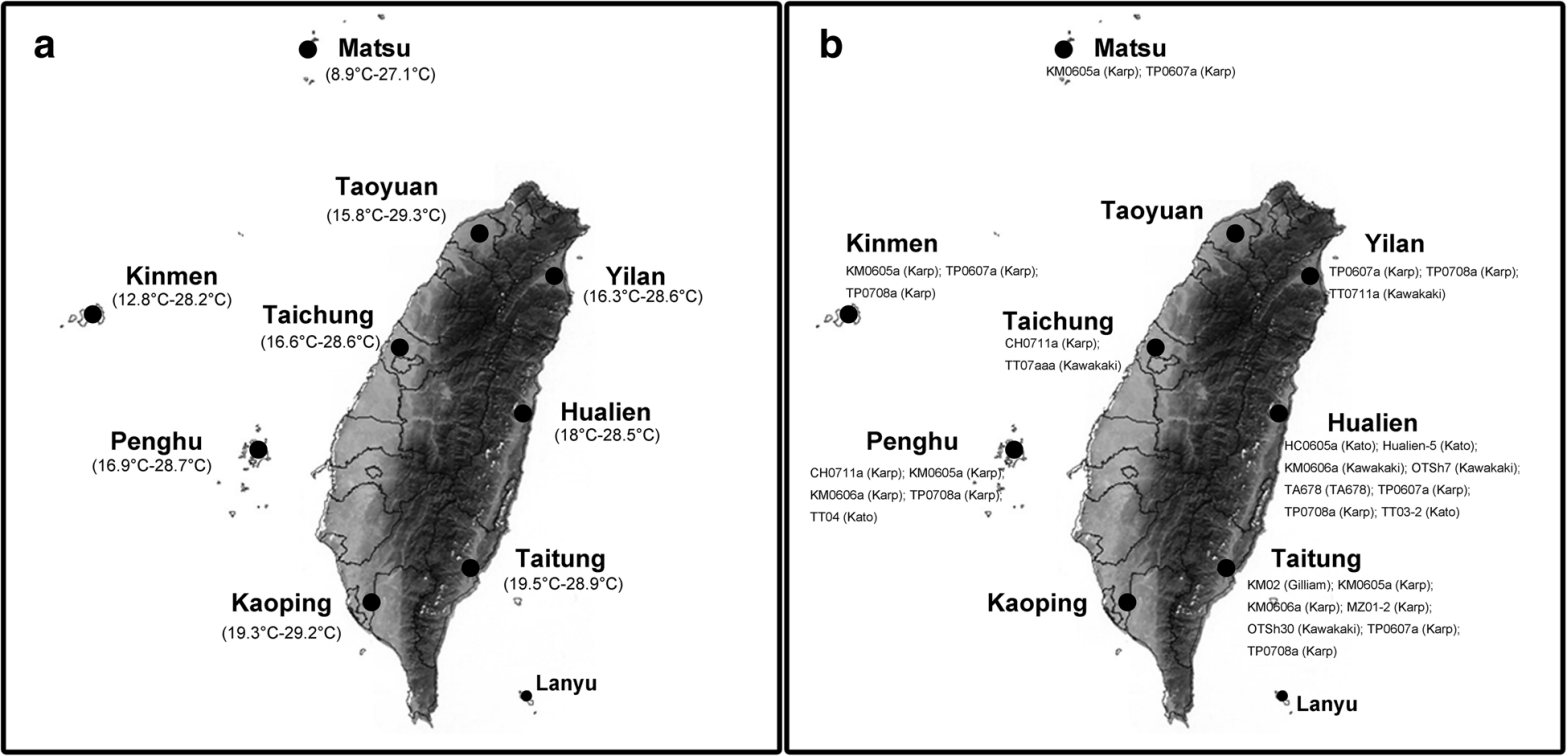
Study sites for the collection and identification of *Orientia tsutsugamushi* (OT) strains in chiggers from small mammal hosts in Taiwan during 2006–2010. **a** mean temperature for the coldest and hottest months (*shown in parenthesis*); **b** OT strains and genotypes (*in parenthesis*) identified in chiggers

From 2006 to 2010, small mammals (rodents and shrews) were trapped in different parts of Taiwan, including eastern (Yilan, Hualien, Taitung), western (Taoyuan, Taichung, Kaoping), and main islets near Taiwan (Matsu, Kinmen, Penghu) (Fig. 1). These nine counties vary profoundly in the incidence rate of scrub typhus (2001–2005, 0.2 to 125.2 cases per 100,000 people per year; on-line data, Taiwan National Infectious Disease Statistics System, http://nidss.cdc.gov.tw/). Mean temperature for the hottest month (typically in July in Taiwan) is similar among the nine study sites (27.1 °C–29.3 °C, Taiwan Central Weather Bureau) while mean temperature for the coldest month (typically in January) is more varied (8.9 °C–19.5 °C), with lower January temperature occurring in Matsu (8.9 °C) and Kinmen (12.8 °C) (Fig. 1a). Because scrub typhus prevails mainly in rural areas and occurs outside house [12], trappings were limited to the field but not implemented inside or close to human buildings. At each county, 80 Sherman traps (26.5 × 10 × 8.5 cm) and 80 Taiwan made rodent traps (27 × 16 × 13 cm) were deployed and baited with sweet potato covered with peanut butter. Each county was surveyed for four consecutive nights and surveyed at least twice (Yilan: March and August 2009; Hualien: May and November 2006, April 2009; Taitung: September 2006, June and September 2009; Taoyuan: March and October 2010; Taichung: March 2009, July 2010; Kaoping: February and May 2010; Matsu: March and July 2007; Kinmen: June 2006, January 2007, March 2008; Penghu: May and September 2007). Each night, traps were set up at different locations within the same county to maximize trapping coverage. Trapped small mammals (shrews and rodents) were euthanized with an overdose of Zoletil 50 (Virbac SA, Carros, France), and blood was collected by cardiac puncture. Chiggers recovered from small mammals were preserved in 70 % ethanol and stored at−70 °C until subsequent molecular detection of OT.

### Species identification of chiggers

Chiggers were soaked in deionized water 2–3 times (30 min each), and then slide-mounted in Berlese fluid (Asco Laboratories, Manchester, UK). Chiggers were examined under a light microscope and identified with published keys to full species level based on a combination of characteristics of scutum, gnathosoma, and ventral and dorsal seta following [25, 26].

### Immunofluorescent antibody assay (IFA)

Seroprevalence of OT exposure in rodents was assessed following [17]. Briefly, each rodent serum sample was diluted 1:40 in phosphate-buffered saline (PBS), applied to slides coated with OT antigens (Gu-Yuan Biotech. Ltd., Taiwan), and mixed by pipetting with fluorescein isothiocyanate–goat anti-mouse immunoglobulin G (IgG) + A + M (H + L; Zymed Laboratories, Inc., San Francisco, CA) diluted 1:40 in PBS. Shrew serum was not assessed to avoid false negativity due to our application of anti-mouse but not anti-shrew secondary antibodies. We chose 1:40 as the cutoff titer because this is the same criterion used for confirmation of human cases of scrub typhus in Taiwan by the Taiwan Centers for Disease Control. OT antigen slides allowed simultaneous screening of three strains (Kato, Karp, and Gilliam). Serum samples were scored negative for OT when all three strains were negative; if any strains yielded positive results, the sample was recorded as positive.

### Detection of OT in chiggers with nested PCR

Pools of chiggers (largely with 100 chiggers for each pool to retrieve enough DNA) were detected for OT infections with nested polymerase chain reaction (PCR) following [27]. This method targeted well conserved DNA corresponding to a 56-kDa type specific antigen located on the OT outer membrane. Primers for the 1st stage PCR were 56 kDa-1 F: 5’-AGAATCTGCTCGCTTGGATCCA-3’ and 56 kDa-1R: 5’-ACCCTATAGTCAATACCAGCACAA-3’ and for the 2nd stage: 56 kDa-2 F: 5’-GAGCAGAGATAGGTGTTATGTA-3’ and 56 kDa-2R: 5’-TATTCATTATAGTAGGCTGA-3’. Laboratory Karp and Gilliam strains and deionized water were used as positive and negative controls, respectively. The PCR products were separated by electrophoresis in 3 % NuSieve and 1.0 % agarose gels, stained with ethidium bromide, and identified under UV fluorescence. The PCR products were purified with a QIA quick Gel Extraction Kit (Qiagen GmbH, Hilden, Germany) and then sequenced twice in each direction. DNA nucleotide sequences were assessed with the Basic Local Alignment Search Tool (www.ncbi.nlm.nih.gov) for any resemblance to known OT strains. Because of the minute size of chiggers, identification of chiggers to full species requires mounting specimens with the use of Berlese fluids, which destroys DNA material and renders the detection of OT unlikely. Chigger pools were thus selected randomly irrespective of species identity.

### Incidence rate of scrub typhus

Information on human cases of scrub typhus, a notifiable diseases in Taiwan, was retrieved from the Taiwan National Infectious Disease Statistics System (http://nidss.cdc.gov.tw/) maintained by Taiwan Centers for Disease Control. Details on presumptive location of infections and the date for the onset of diseases were available from the database. We enumerated the number of human cases in the towns (a county includes several towns) where small mammals were trapped and included only cases reported during the period when trapping was implemented. Due to the relatively few human cases of scrub typhus in most of the towns, the period during which human cases were tallied included not only the month when small mammals were trapped but also its preceding and following month (totaling three months; this will increase human cases and thus statistical power). Population size for each town when trapping was implemented was obtained from the Department of Statistics of the Taiwan Ministry of the Interior. Towns were aggregated into counties for the calculation of incidence rate, which was defined as number of human cases per 100000 residents per month.

### Statistical analysis

Correlation analyses were applied when studying association between loads and prevalence of chigger infestations, OT seropositivity rate in rodents, OT positivity rate in chiggers, and human incidence rate of scrub typhus. Because normality could not be achieved for most of the analyses even after data transformation, non-parametric Spearman rank correlations were used for all of the correlational studies.

### Ethical considerations

This study was implemented with the permission of Taiwan Centers for Disease Control and all trapping and handling procedures met Taiwanese legal requirements.

## Results

### Small mammal trapping

A total of 1,285 small mammals were trapped, including one species of shrew (*Suncus murinus*) and 10 species of rodents (Table 1). Among all species, *Rattus losea* was the most abundant (48.7 % of total captures), followed by *S. murinus* (21.2 %) and *Mus caroli* (11.2 %). *R. losea* was also the most widely distributed species, occurring in all nine counties. On the contrary, *Rattus tanezumi* were trapped only in Lanyu Island (Orchid Island) in Taitung county, *Rattus exulans* only in Hualien county, and the squirrel *Callosciurus erythraeus* only in Yilan county (Table 1).

**Table 1:** Prevalence (%) and loads of chigger infestations, seroprevalence (%) of *Orientia tsutsugamushi* (OT) exposure, and OT positivity rate (%) in chiggers among small mammal hosts in Taiwan during 2006–2010

Host species	No. of captures (% of total)	No. of counties	Prevalence (%) of chiggers	Mean # of chiggers/host (±SE)^a^	Total chiggers (% of all)	Seroprevalence (no. of assayed)	OT positivity rate (no. of assayed)
Shrews							
Suncus murinus	272 (21.2)	8	16.2	12.8 ± 3.3	3,469 (2.7)	NA	10.5 (19)
Rodents							
Apodemus agrarius	24 (1.9)	4	54.2	82.3 ± 18.2	1,975 (1.5)	57.9 (19)	0 (13)
Bandicota indica	83 (6.5)	6	45.8	51.7 ± 11.2	4,288 (3.3)	35.8 (81)	51.9 (27)
Callosciurus erythraeus	2 (0.2)	1	0	0	0 (0)	0 (1)	NA
Mus caroli	144 (11.2)	8	19.4	1.9 ± 0.5	271 (0.2)	16.1 (124)	NA
Mus musculus	47 (3.7)	6	14.9	2.3 ± 1.1	109 (0.1)	56.5 (46)	NA
Niviventer coninga	2 (0.2)	2	0	0	0 (0)	100 (1)	NA
Rattus exulans	26 (2.0)	1	34.6	14.8 ± 4.6	385 (0.3)	15.4 (26)	66.7 (9)
Rattus losea	626 (48.7)	9	61.3	156.9 ± 11.2	98,239 (76.4)	46.2 (520)	54.8 (230)
Rattus norvegicus	11 (0.9)	6	9.1	79.3 ± 79.3	872 (0.7)	20.0 (10)	0 (1)
Rattus tanezumi	48 (3.7)	1	100	394.0 ± 28.5	18,912 (14.7)	91.7 (48)	95.8 (48)
Overall	1,285	9	44.5	100.0 ± 6.2	128,520	43.0 (876)	55.9 (347)

^a^Mean # chiggers/host is calculated across all captures, not just those animals that harboured ticks

### Prevalence and loads of chiggers among small mammal species

Prevalence of chigger infestations among host species varied from 9.1 % to 100 %, disregarding the very few captures of *C. erythraeus* and *Niviventer coninga* (Table 1). All *R. tanezumi* trapped in Lanyu Island were infested with chiggers (100 % prevalence). *R. losea* (61.3 %), *Apodemus agrarius* (54.2 %), and *Bandicota indica* (45.8 %) were also frequently infested (Table 1).

Mean number of chiggers per host varied profoundly among small mammal species, too. *R. tanezumi* had the highest chigger loads (394 ± 28.5, mean ± SE), followed by *R. losea* (156.9 ± 11.2). In comparison, chigger loads were much lower in *M. caroli* (1.9 ± 0.5) and *M. musculus* (2.3 ± 1.1) (Table 1). There was a significant correlation between prevalence and loads of chigger infestations in host species (Spearman rank correlation, *r*_*s*_ = 0.84, d.f. = 10, *P* < 0.005).

A total of 128,520 chiggers were collected from small mammal hosts. 76.4 % of these chiggers were hosted by *R. losea*. This was followed by *R. tanezumi* (14.7 %). Each of the other host species supported <5 % of chiggers (Table 1).

### Chigger species composition among small mammal species

Among the 2,860 identified chiggers this included eight *Leptotrombidium* species, three *Walchia* species, and one *Eutrombicula* species (Table 2). *L. deliense* was the most common species (64.9 % of all identified chiggers). *L. imphalum* (10.8 %), *Walchia pacifica* (7.0 %), *Walchia xishaensis* (4.1 %), *L. pallidum* (4.0 %), and *L. scutellare* (3.7 %) were uncommon while the other six chigger species were rarely observed (Table 2).

**Table 2:** Total number of chiggers of each species (and % of total chiggers) recovered from small mammal hosts in Taiwan during 2006–2010

Host species	*L. akamushi*	*L. deliense*	*L. imphalum*	*L. kawamurai*	*L. pallidum*	*L. rubellum*	*L. scutellare*	*L. yui*	*W. chinesis*	*W. pacifica*	*W. xishaensis*	*E. wichmanni*	Unknown	Total chiggers
Shrews														
Suncus murinus	0 (0)	179 (84.4)	1 (0.5)	24 (11.3)	0 (0)	0 (0)	0 (0)	0 (0)	0 (0)	0 (0)	0 (0)	0 (0)	8 (3.8)	212
Rodents														
Apodemus agrarius	1 (0.6)	56 (32.0)	111 (63.4)	0 (0)	0 (0)	0 (0)	0 (0)	0 (0)	0 (0)	0 (0)	0 (0)	0 (0)	7 (4.0)	175
Bandicota indica	0 (0)	407 (66.7)	1 (0.2)	0 (0)	0 (0)	0 (0)	0 (0)	0 (0)	0 (0)	197 (32.3)	0 (0)	0 (0)	5 (0.8)	610
Mus caroli	0 (0)	10 (37.0)	10 (37.0)	0 (0)	0 (0)	0 (0)	0 (0)	0 (0)	0 (0)	0 (0)	0 (0)	0 (0)	7 (25.9)	27
Rattus exulans	0 (0)	15 (20.5)	41 (56.2)	1 (1.4)	0 (0)	0 (0)	0 (0)	0 (0)	0 (0)	2 (2.7)	0 (0)	0 (0)	14 (19.2)	73
Rattus losea	0 (0)	1091 (65.8)	146 (8.8)	4 (0.2)	113 (6.8)	5 (0.3)	106 (6.4)	14 (0.8)	2 (0.1)	0 (0)	112 (6.8)	0 (0)	64 (3.9)	1657
Rattus norvegicus	0 (0)	10 (100)	0 (0)	0 (0)	0 (0)	0 (0)	0 (0)	0 (0)	0 (0)	0 (0)	0 (0)	0 (0)	0 (0)	10
Rattus tanezumi	0 (0)	89 (92.7)	0 (0)	0 (0)	0 (0)	0 (0)	0 (0)	0 (0)	0 (0)	0 (0)	5 (5.2)	2 (2.1)	0 (0)	96
Overall (% of all)	1 (0.03)	1857 (64.9)	310 (10.8)	29 (1.0)	113 (4.0)	5 (0.2)	106 (3.7)	14 (0.5)	2 (0.07)	199 (7.0)	117 (4.1)	2 (0.07)	105 (3.7)	2860

*L. akamushi* (*Leptotrombidium akamushi*); *L. deliense* (*Leptotrombidium deliense*); *L. imphalum* (*Leptotrombidium imphalum*); *L. kawamurai* (*Leptotrombidium kawamurai*); *L. pallidum* (*Leptotrombidium pallidum*); *L. rubellum* (*Leptotrombidium rubellum*); *L. scutellare* (*Leptotrombidium scutellare*); *L. yui* (*Leptotrombidium yui*); *W. chinesis* (*Walchia chinesis*); *W. pacifica* (*Walchia pacifica*); *W. xishaensis* (*Walchia xishaensis*); *E. wichmanni* (*Eutrombicula wichmanni*)

*L. deliense* was the most dominant chigger species in small mammal hosts except in *A. agrarius* and *R. exulans*, which harboured more *L. imphalum* than *L. deliense* (*M. caroli* excluded for its small number of samples). A moderate proportion of chiggers (32.3 %) in *B. indica* also comprised of *W. pacifica* (Table 2).

### Seroprevalence among small mammal species

Seropositivity rate for the 876 rodents assayed for OT exposure was 43.0 %. Positivity rate differed significantly among rodent species, ranging from 15.4 % to 91.7 % when *C. erythraeus* and *N. coninga* were excluded (both with small sample size of one). There was a tendency for rodent species with higher chigger loads to have higher seropositivity rate (*r*_*s*_ = 0.64, d.f. = 7, *P* = 0.08) except for *M. musculus*, which had low chigger load (2.3 chiggers per mouse) but with moderate seropositivity rate (56.5 %) (*r*_*s*_ = 0.89, d.f. = 6, *P* < 0.01 after *M. musculus* excluded from analysis). Accordingly, seropositivity rate was higher in *R. tanezumi* (91.7 %), *R. losea* (46.2 %) and *A.agrarius* (57.9 %) and was lower in *M. caroli* (16.1 %) and *R. exulans* (15.4 %) (Table 1).

### PCR positivity rate and OT strains identified in chiggers

A total of 347 pools of chiggers were detected for 56 kDa-PCR positivity, with a positivity rate of 55.9 % (Table 1). Both *R. tanezumi* and *R. losea* that had high chigger loads and seropositivity rates also harboured chiggers with higher OT positivity rate (95.8 % and 54.8 %, respectively). In comparison, *R. exulans* carried chiggers with moderate positivity rate (66.7 %) even with low chigger loads and seropositivity rate while *A. agrarius* had moderate chigger loads and seropositivity rate but with very low positivity rate in chiggers (0 %) (Table 1). There was no significant correlation between chigger loads and OT positivity rate in chiggers (*r*_*s*_ = 0.27, d.f. = 6, *P* > 0.05); likewise, there was no significant correlation between seropositivity rate and OT positivity rate (*r*_*s*_ = 0.17, d.f. = 5, *P* > 0.05).

A total of 15 OT strains belonging to five genotypes were identified in the 107 successfully sequenced samples (Table 3). TP0607a was the most common strain (33.6 % of samples), followed by KM0606a (15.9 %), TP0708a (11.2 %), KM0605a (10.3 %), and MZ01-2 (6.5 %). Each of the remaining strains comprised <5 % of all sequenced samples (Table 3). The five genotypes, starting from the commonest, were Karp (66.4 %), Kawasaki (24.3 %), Kato (7.5 %), Gilliam (0.9 %), and TA678 (0.9 %).

**Table 3:** Strains or closely related strains of *Orientia tsutsugamushi* (OT) identified in chiggers collected from small mammals in Taiwan during 2006–2010

OT strains	Similarity to the closest strain (length of nucleotide)	Genotype	No. of strains (% of total)	GenBank accession no.	Sites (no. of identification)
CH0711a	98 % (145/148)	Karp	5 (4.7)	GQ332749	Penghu (1); Taichung (4)
HC0605a	100 % (102/102)	Kato	2 (1.9)	GQ332761	Hualien (2)
Hualien-5	100 % (102/102)	Kato	3 (2.8)	AY714316	Hualien (3)
KM02	100 % (123/123)	Gilliam	1 (0.9)	GU120147	Taitung (1)
KM0605a	100 % (145/145)	Karp	11 (10.3)	GQ332742	Kinmen (3); Matsu (1); Penghu (6); Taitung (1)
KM0606a	100 % (126/126)	Kawasaki	17 (15.9)	GQ332760	Hualien (8); Penghu (7); Taitung (2)
MZ01-2	100 % (126/126)	Karp	7 (6.5)	GU120156	Taitung (7)
OTSH7	100 % (126/126)	Kawasaki	3 (2.8)	KF777269	Hualien (3)
OTSh30	100 % (108/108)	Kawasaki	1 (0.9)	KF777288	Taitung (1)
TA678	95 (105/111)	TA678	1 (0.9)	U19904	Hualien (1)
TP0607a	100 (126/126)	Karp	36 (33.6)	GQ332744	Hualien (10); Kinmen (11); Matsu (3); Taitung (3); Yilan (9)
TP0708a	100 (126/126)	Karp	12 (11.2 %)	GQ332745	Hualien (7); Kinmen (1); Penghu (1); Taitung (2); Yilan (1)
TT03-2	100 (102/102)	Kato	1 (0.9)	GU120169	Hualien (1)
TT04	100 (102/102)	Kato	2 (1.9)	GU120170	Penghu (2)
TT0711a	100 (126/126)	Kawasaki	5 (4.7)	GQ332742	Taichung (1); Yilan (4)

### Geographical variation in incidence rate of scrub typhus, chigger infestations, and OT occurrence

Because small mammal species varied in the extent of chigger infestations (Table 1), geographical variation would ideally be compared based on a single host species to control for any spatial variation in the composition of local small mammal species. *R. losea* was thus selected for comparison because of its abundance in all study sites.

Incidence rate of scrub typhus (cases per 100000 residents per month) varies significantly among the nine counties, ranging from zero (Kaoping, Taichung, and Yilan) to 12.13 (Penghu) (Table 4). There was also a profound geographical variation, with *R. losea* as the proxy, in the chigger loads, prevalence of chigger infestations, seropositivity rate, and OT positivity rate in chiggers (Table 4). Counties with higher incidence rate also harboured *R. losea* with higher chigger loads (*r*_*s*_ = 0.75, d.f. = 8, *P* < 0.05), higher prevalence of chigger infestations (*r*_*s*_ = 0.73, d.f. = 8, *P* < 0.05), and higher seropositivity rate (*r*_*s*_ = 0.80, d.f. = 8, *P* < 0.05). There was nevertheless no correlation between incidence rate and OT positivity rate in chiggers (*r*_*s*_ = −0.23, d.f. = 6, *P* > 0.05).

**Table 4:** Prevalence (%) and loads of chigger infestations, seroprevalence (%) of *Orientia tsutsugamushi* (OT) exposure, and OT positivity rate (%) in chiggers on *Rattus losea* at different sites in Taiwan during 2006–2010

Study site	No. of *R. losea* captures	Prevalence (%) of chiggers	Mean # of chiggers/host (±SE)^*a*^	Seroprevalence (no. of assayed)	OT positivity rate (no. of assayed)	Incidence rate of scrub typhus^*b*^
Eastern Taiwan						
Yilan	91	67.0	72.9 ± 8.5	55.6 (72)	71.2 (52)	0
Hualien	41	95.1	359.0 ± 44.1	80.5 (41)	71.8 (39)	0.65
Taitung	20	95.0	158.7 ± 30.2	63.2 (19)	40.0 (15)	2.18
Western Taiwan						
Taoyuan	116	0	0	0.9 (116)	NA	0.05
Taichung	33	6.1	2.7 ± 2.3	3.2 (31)	50.0 (2)	0
Kaoping	44	0	0	0 (44)	NA	0
Offshore islands						
Matsu	57	94.7	324.5 ± 34.4	60.0 (55)	9.5 (42)	6.44
Kinmen	168	91.1	145.4 ± 15.2	77.9 (86)	65.8 (38)	7.31
Penghu	56	100	548.4 ± 75.2	94.6 (56)	59.5 (42)	12.13

^*a*^Mean # chiggers/host is calculated across all captures, not just those animals that harboured ticks

^*b*^Number of human cases per 100000 residents per month

### Geographical variation in chigger species composition

*L. deliense* was the dominant chigger species (>70 % of total chiggers) in most study sites (disregarding Kaoping and Taoyuan for collecting no or very few chiggers). However, *L. imphalum* and *L. pallidum* were more common in Hualien and Matsu, respectively (Table 5). Besides, there was a clear seasonal variation in the occurrence of chigger species in Matsu. *L. deliense* and *Leptotrombidium kawamurai* were only collected in the summer (July) while *L. pallidum*, *L. scutellare*, and *Leptotrombidium yui* were only observed in the early spring (March). Similarly, in Kinmen, *L. deliense* was found exclusively in the summer (June) and *L. scutellare* solely in the winter (January).

**Table 5:** Number of chiggers of each species (and % of total chiggers) recovered from different study sites in Taiwan during 2006–2010

Host species	*L. akamushi*	*L. deliense*	*L. imphalum*	*L. kawamurai*	*L. pallidum*	*L. rubellum*	*L. scutellare*	*L. yui*	*W. chinesis*	*W. pacifica*	*W. xishaensis*	*E. wichmanni*	Unknown	Total chiggers
Eastern Taiwan														
Yilan	0 (0)	387 (96.8)	4 (1.0)	0 (0)	0 (0)	0 (0)	0 (0)	2 (0.5)	0 (0)	6 (1.5)	0 (0)	0 (0)	1 (0.3)	400
Hualien	1 (0.2)	210 (36.1)	296 (50.9)	5 (0.9)	0 (0)	4 (0.7)	0 (0)	0 (0)	0 (0)	3 (0.5)	0 (0)	0 (0)	63 (10.8)	582
Taitung	0 (0)	457 (72.9)	9 (1.4)	0 (0)	0 (0)	1 (0.2)	0 (0)	0 (0)	0 (0)	0 (0)	117 (18.7)	2 (0.3)	41 (6.5)	627
Western Taiwan														
Taoyuan	0 (0)	0 (0)	0 (0)	0 (0)	0 (0)	0 (0)	0 (0)	0 (0)	0 (0)	16 (100)	0 (0)	0 (0)	0 (0)	16
Taichung	0 (0)	423 (70.9)	0 (0)	0 (0)	0 (0)	0 (0)	0 (0)	0 (0)	0 (0)	174 (29.2)	0 (0)	0 (0)	0 (0)	597
Kaoping	0 (0)	0 (0)	0 (0)	0 (0)	0 (0)	0 (0)	0 (0)	0 (0)	0 (0)	0 (0)	0 (0)	0 (0)	0 (0)	0
Offshore islands														
Matsu	0 (0)	76 (31.4)	0 (0)	24 (9.9)	113 (46.7)	0 (0)	17 (7.0)	12 (5.0)	0 (0)	0 (0)	0 (0)	0 (0)	0 (0)	242
Kinmen	0 (0)	105 (53.6)	0 (0)	0 (0)	0 (0)	0 (0)	89 (45.4)	0 (0)	2 (1.0)	0 (0)	0 (0)	0 (0)	0 (0)	196
Penghu	0 (0)	199 (99.5)	1 (0.5)	0 (0)	0 (0)	0 (0)	0 (0)	0 (0)	0 (0)	0 (0)	0 (0)	0 (0)	0 (0)	200

*L. akamushi* (*Leptotrombidium akamushi*); *L. deliense* (*Leptotrombidium deliense*); *L. imphalum* (*Leptotrombidium imphalum*); *L. kawamurai* (*Leptotrombidium kawamurai*); *L. pallidum* (*Leptotrombidium pallidum*); *L. rubellum* (*Leptotrombidium rubellum*); *L. scutellare* (*Leptotrombidium scutellare*); *L. yui* (*Leptotrombidium yui*); *W. chinesis* (*Walchia chinesis*); *W. pacifica* (*Walchia pacifica*); *W. xishaensis* (*Walchia xishaensis*); *E. wichmanni* (*Eutrombicula wichmanni*)

### Geographical variation in OT strains and genotypes

TP0607a and TP0708a were the most widely distributed OT strain, followed by KM0605a (Table 3). Among the five OT genotypes, Karp was identified in all study sites (excluding Taoyuan and Kaoping where no chiggers were detected for OT), and Kawasaki was also not uncommon, especially in eastern Taiwan (Fig. 1b). Eastern Taiwan also harboured the most diverse OT strains and genotypes, especially Hualien and Taitung (Fig. 1b).

## Discussion

In this study, *R. losea* was found to be the most abundant and widespread rodent species in Taiwan; it also hosted the highest proportion of chiggers among all small mammal species. *L. deliense* was identified as the dominant chigger species in Taiwan but its occurrence was limited to certain seasons (mainly summer) in some study sites (Kinmen and Matsu).

*R. losea* commonly occurs in agricultural fields and grasslands in lowland Taiwan, including most islets near Taiwan ([28]; this study, data not shown). In Kinmen, this species was sometimes classified as *Rattus flavipectus* (e.g. [21, 24]), but it was recently confirmed to be *R. losea* based on molecular evidence [29]. We have found that *R. losea* hosted the majority of chiggers (76.4 %) and OT positivity rate in these chiggers was higher than those collected from most other host species. Moreover, our previous study revealed a higher feeding success of chiggers on *R. losea* than on the other two main host species in eastern Taiwan [18]. *R. losea* thus deserves special attention when rodent control is envisaged as a viable strategy for lowering the burden of scrub typhus. Because *R. losea* rarely occurs in forests (including secondary forests), which is instead inhabited by *N. coninga* [30, 31], preserving lowland forests in Taiwan could potentially mitigate the risks to scrub typhus through a reduction in the occurrence of *R. losea*. This will also depend on the relative importance of *N. coninga* as hosts of chiggers, which to our best knowledge, has never been intensively investigated before. But the low population density of *N. coninga* in Taiwan [31] suggests that *R. losea* might be a better host of chiggers than *N. coninga* in lowland Taiwan.

Among all vertebrate hosts, *R. tanezumi* had the highest loads and prevalence of chigger infestations, OT seropositivity rate in rodents, and OT positivity rate in chiggers. This rodent species was recognized as *Rattus rattus* before (e.g. [24]); however, *R. rattus* in Taiwan should instead be *R. tanezumi* [32]. *R. tanezumi* commonly occurs near human dwellings in Taiwan, but in this study, it was trapped only in Lanyu Island. This is due to the fact that we deployed traps in the field but not inside or close to human buildings and Lanyu Island is unusual in that *R. tanezumi* can be easily found in grassland and forest in Lanyu [33]. High chigger infestation and OT positivity rate in *R. tanezumi* should reflect a high risk to scrub typhus in Lanyu. Indeed, seropositivity rate for scrub typhus was extremely high (100 %) when children in Lanyu reached 7 years old [34]. Why Lanyu Island shelters abundant chiggers remains unclear and should warrant further investigation.

*L. deliense* is the most dominant and widespread vector of scrub typhus in the tropics of Asia and southwest Pacific islands, including Malaysia, New Guinea, Philippine, Thailand and many other countries [11]. In this study, *L. deliense* was also found to be the most common and widely distributed chigger species in Taiwan. Because of the high infectivity rate of OT in *L. deliense* and *L. deliense* can readily bite humans [26], the commonness of this chigger species in Taiwan indicates that people in Taiwan could be at high risk of scrub typhus infection. However, in Matsu and Kinmen, two offshore islands with lower winter temperature than the other study sites (Fig. 1a), *L. deliense* occurred only in the summer. In Kinmen, this species was replaced by *L. scutellare* in the winter. Likewise, *L. pallidum*, *L. scutellare*, and *L. yui* appeared in the cooler season in Matsu. Both *L. pallidum* and *L. scutellare* have been found in Japan and South Korea and occur mainly from fall to early spring [11, 35, 36]. In Yunnan of China, *L. deliense* is distributed at lower latitudes and *L. scutellare* at higher latitudes [37]. In South Korea, *L. scutellare* occurs primarily in the southern part of the country while *L. pallidum* in found more in northern regions [36]. The studies in China, Japan, and South Korea indicate that relative to *L. deliense*, *L. pallidum* and *L. scutellare* live in cooler environments, and *L. pallidum* can survive at further lower temperatures than *L. scutellare*. This agrees with our observation that *L. pallidum* and *L. scutellare* occurred only during cooler seasons and *L. pallidum* was distributed in Matsu rather than Kinmen, where winter temperature is lower in Matsu (8.9 °C) than in Kinmen (12.8 °C).

For the past 10 years (2005–2014), a total of 689 cases of scrub typhus were recorded in Kinmen (on-line data, accessed 26th July 2015, Taiwan National Infectious Disease Statistics System, http://nidss.cdc.gov.tw/). Most of these human cases (671 cases, or 97.4 % of all cases) occurred from late spring (May) to mid-fall (October). Likewise, 92.4 % of the 144 cases of scrub typhus from 2005 to 2014 in Matsu happened between June and October. Seasonal occurrence of human cases in Kinmen and Matsu roughly matched the seasonal appearance of *L. deliense*, suggesting that *L. deliense* was the main vector in both islands; our previous study in Kinmen [21] arrived at a similar conclusion. It is unexpected, however, that there were very few human cases from late fall to early spring in Kinmen and Matsu when *L. pallidum* or *L. scutellare* appeared because both species have been proven to be vectors of scrub typhus in Japan and South Korea [11, 35, 36]. The causes for such a lack of cases during the winter are unknown, and could be due to less human outdoor activity during the winter or other not yet recognized reasons. However, the number of cases peaking during the warm season implies that global warming might increase incidence of scrub typhus or lengthen the period during which scrub typhus prevails in Kinmen and Matsu. Monitoring abundance of main chigger vectors and their association with meteorological factors and human incidence is thus needed.

Besides incidence rate of scrub typhus, there also existed a profound geographical variation in loads and prevalence of chigger infestations in rodents (Table 4). The reasons for such spatial variation have previously been explored but conclusive remarks can only be made after moving beyond simple correlational studies. Incidence rate of scrub typhus on the main island of Taiwan was elevated in districts with a higher proportion of dry-field farmers in the population, a higher normalized difference vegetation index and lower mean annual temperature [15]. It was further revealed that incidence rate also increased with the proportion of land that contained mosaics of cropland and natural vegetation, which may be because a mixture of crop and vegetation habitats are ideal for rodents by providing easy access to abundant food from nearby vegetative shelters [16]. Chiggers could also proliferate in this kind of favourable habitat with abundant rodent hosts and natural vegetation that helps provide moist terrain essential for chigger survival [27]. Indeed, lower chigger infestations occur in highly populated western Taiwan where less natural vegetation survives intensive human exploitation.

With *R. losea* as a surrogate, we found that incidence rate of scrub typhus was positively correlated with chigger loads, prevalence of chigger infestations and seropositivity rate but not OT positivity rate in chiggers. In addition, more than half (>0.73^2^ = 0.533) of the variation in incidence rate can be accounted for by chigger loads, chigger prevalence, or seropositivity rate. This is despite the fact that human infection to scrub typhus is a product of several factors, at least including number of questing chiggers (which may or may not be correlated with loads of chiggers in vertebrate hosts), infectivity rate of OT in questing chiggers, and intensity of human activity in infested areas. Such significant association is useful because in our experience, surveying questing chiggers, including the method such as black plates, could be very inefficient particularly when few chiggers exist. Our study indicates that instead, by examining hosts, each of these indices (loads and prevalence of chigger infestations on hosts or seropositivity rate of hosts) can be a useful proxy for relative human risks to scrub typhus, as has been found in South Korea where incidence rate increased with the load of chiggers on rodents [38].

## Conclusions

Scrub typhus is emerging in many regions and whether the rapid increase in incidence is associated with global warming or alteration of habitat is still unknown. Our study reveals *R. losea* as the primary host for chiggers and there exists a geographical and seasonal variation in chigger species in Taiwan. We argue that only with a solid knowledge on chigger vectors and their vertebrate hosts can we start to predict the consequence of environmental change for human risks to scrub typhus.
